# Interprofessional education: tips for design and implementation

**DOI:** 10.1186/s12909-020-02286-z

**Published:** 2020-12-03

**Authors:** Christie van Diggele, Chris Roberts, Annette Burgess, Craig Mellis

**Affiliations:** 1 The University of Sydney, Faculty of Medicine and Health, The University of Sydney, Edward Ford Building A27, Sydney, NSW 2006 Australia; 2 The University of Sydney, Faculty of Medicine and Health, Sydney Health Professional Education Research Network, The University of Sydney, Sydney, Australia; 3 The University of Sydney, Faculty of Medicine and Health, Sydney Medical School – Education Office, The University of Sydney, Sydney, Australia; 4 The University of Sydney, Faculty of Medicine and Health, Sydney Medical School - Central Clinical School, The University of Sydney, Sydney, Australia

**Keywords:** Interprofessional education, Multidisciplinary, Facilitation, Teamwork, Collaboration

## Abstract

Interprofessional education (IPE) is a critical approach for preparing students to enter the health workforce, where teamwork and collaboration are important competencies. IPE has been promoted by a number of international health organisations, as part of a redesign of healthcare systems to promote interprofessional teamwork, to enhance the quality of patient care, and improve health outcomes. In response, universities are beginning to create and sustain authentic and inclusive IPE activities, with which students can engage. A growing number of health professionals are expected to support and facilitate interprofessional student groups. Designing interprofessional learning activities, and facilitating interprofessional groups of students requires an additional layer of skills compared with uniprofessional student groups. This article outlines the key points for planning and practicing interprofessional facilitation within the classroom and clinical setting.

## Background

The World Health Organisation (WHO) Framework for Action on Interprofessional Education and Collaborative Practice (2010), states that “*Interprofessional education occurs when two or more professionals learn about, from and with each other to enable effective collaboration and improve health outcomes”* [1]. In an increasingly complex healthcare system, members of the health service delivery team need to collaborate with each other to accomplish common goals to improve the patient’s experience and outcomes [2, 3]. There is international agreement that health professional students should be prepared for practice by experiencing Interprofessional Education (IPE). Many international health organisations have promoted IPE in the context of an aging population, limited financial resources, and the recognition of a need to redesign the healthcare system to improve teamwork between disciplines, enhance quality of patient care, and improve health outcomes [2, 4]. Universities have been challenged to create and maintain authentic IPE activities that are inclusive of all cohorts [4]. It is critical for health professional students and graduates to engage with the IPE opportunities that they are presented with across various clinical environments at the level of pre-qualification and pre-registration [5].

Despite the abundance of IPE reviews targeting staff, there is a paucity of guidance for students wishing to actively engage in IPE activities as a facilitator. This paper provides health professional students and junior health professionals with strategies for planning, designing and facilitating interprofessional groups of students within formal classroom and informal clinical settings.

### Interprofessional education: what and why?

Most health professional education is uniprofessional, where the goal is developing the depth of disciplinary knowledge necessary for the newly qualified graduate to be prepared for practice. Learning from and with other health professional students can occur in many environments, including large classes, small group tutorials, simulation and the clinical setting. Meeting the learning goals of IPE can occur through planned collaborative learning experiences, but also through the unplanned encounters where students are co-located in clinical placements. Institutions that support interprofessional collaboration work on developing and maintaining effective interprofessional working relationships with learners, practitioners, patients/clients, families and communities [6]. The leadership teams working alongside healthcare teams embrace IPE as a requirement of health professional practice, that is key to delivering good healthcare [7, 8].

### Interprofessional competency development

The learning goals of any IPE activity are best drawn from existing interprofessional competency frameworks. There are several of these to choose from, including the Canadian Interprofessional Health Collaborative [6] and Interprofessional Education Collaborative (2016) [9]. The core competencies for IPE can be summarised into five themes [9]:

1. Roles and responsibilities
2. Ethical practice
3. Conflict resolution
4. Communication
5. Collaboration and teamwork

One or more of these themes should be considered as an outcome when designing an interprofessional activity and where possible matched to an assessment task [9, 15, 16]. Table 1 summarises some of the key issues underpinning each of these five themes to take into account when designing and/or facilitating or providing feedback on teamwork activities [10–16].

**Table 1:** Common themes of IPE

Roles and responsibilities	No single health profession is capable of meeting all patient needs, fuelling the desire to collaborate. Many professionals understand that the more they ‘know’ about other professional’s roles, the more they will know how to operate and function as a team [10]. Tension between individuals arises from people stepping on other’s toes, as ‘role blurring’ results in team members risking conflict, team dysfunction, and burnout. Clear boundaries and role descriptions can assist in finding a balance, just as focusing on a patient’s needs can reduce many perceived professional boundaries [10].
Ethical practice	Ethical practice within healthcare is classed as the standards of practice and responsibilities professionals hold with their patients and colleagues. Heavily reliant on collaborative, team practice and moral obligation, health professionals are required to make complex ethical decisions as a team [11].
Conflict resolution	Health professionals are encouraged to actively engage with other professionals and patients in a positive manner, addressing disagreements in a constructive manner as they arise. Conflicts can be resolved by identifying and addressing specific areas of disagreement and by establishing safe environments and structures to express differing opinions and viewpoints [6].
Communication	Communication is a central concept to many interprofessional frameworks, functioning as the core process through which collaboration occurs [7]. Poor information transfer is closely linked to poor patient outcomes, and potential harm [10]. Communication exists on individual and group levels and occurs in both formal (meetings, patient records) and informal ways (emails, passing comments). Organisations can foster the use of tools such as ISBAR (a clinical handover design using “Introduction, Situation, Background, Assessment Recommendation”), and protocols to assist in effective workplace communication - particularly patient handover [7, 12]. Skilful communication can enable individuals to overcome differences in opinion and negotiate reaching consensus [12]. It is useful to adjust language and terminology used to suit the intended audience and team members. Questioning should also be adjusted to approach situations from different perspectives [10].
Collaboration and teamwork	Collaborative practice is the central component of IPE, for without this, teams cannot effectively function [13]. Learning which includes regular interactions in interprofessional teams, has been shown to produce positive change in student perceptions towards IPE, regardless of the type of activity [14]. Collaborative practice not only refers to the health professionals working together, but also with the patients, their families and the community to provide effective healthcare [1]. Evidence suggests that collaborative practice can improve access to health-services, health outcomes, patient care and safety [1].

### Preparing for Interprofessional learning activities

In IPE, there are opportunities for both formal and informal learning experiences. While informal experiences can assist students in their communication and confidence in their area of expertise, structured formal experiences are more beneficial for beginning students to scaffold their learning [2]. For example, one might compare the medical and pharmacy student experience of participating in a pharmaceutical ward round (informal), with their experience of patient management planning in an interprofessional student-led clinic (formal). Participation in IPE as a formal, planned learning experience works towards the goal of developing students’ attitudes, knowledge, skills and professional behaviours [2].

When designing interprofessional activities, constructive alignment ensures learning outcomes are directly aligned with the activity, and to relevant assessment tasks. This must be made evident to the participating students at the start of an activity. Facilitating interprofessional groups of students is similar in theory and practice to that of uniprofessional groups of students, with the fundamentals remaining the same in planning and activity design. However, literature suggests that facilitators need to adjust their teaching strategies to interact and direct student learning for different professions [17]. In turn this requires more rigorous preparation and guidance [17]. Table 2 displays items to consider when planning learning activities for uniprofessional groups in comparison to interprofessional groups of students [17, 18].

**Table 2:** Comparison of student group activities

	Uniprofessional student group	Interprofessional student group
**Environment**	Various settings e.g. lecture theatres, classrooms, clinical settings, informal conversation.	Various settings e.g. lecture theatres, classrooms, clinical settings, informal conversation.
**Team dynamics**	Students may have pre-existing friendships, and an understanding of each others’ knowledge and skill levels.	Increased chance of miscommunication due to different disciplines and terminology being involved. Possibility of hierarchical issues.
**Grouping**	Grouped according to experience, mixed or random grouping. Often pre-determined grouping by the facilitator is preferable.	Ensure a mixed student group i.e. students should work with students from different disciplines in their groups. Pre-determined grouping by the facilitator is preferable.
**Facilitators**	Most often a professional of the discipline being taught e.g. Nurse educating nursing students.	Should represent the various disciplines of students being taught e.g. if nursing, medical and pharmacy students are present, facilitators should be from those disciplines.
**Activity design**	Individual and group activities should be included.	Majority of activities should be group based to ensure students are gaining the most of the interprofessional experience.
**Assessment**	Assessed well and often. Assessment types include exams, Objective Structured Clinical Examinations (OSCEs), teamwork, Team-based learning (TBL), essays, etc.	Professional skills based assessment e.g. communication. Peer Assessment.

### Facilitating interprofessional education

Representatives including students from various disciplines should contribute to IPE programs and activities through joint planning, the investment of time, accountability, and a commitment to the facilitation of interprofessional learning [19]. Role modelling of ‘interprofessional leadership’ by facilitators, allows students to witness the collaborative nature of joint leadership, promoting trust and acceptance of interprofessional practice. It is important to facilitate the scaffolding of student learning, and support students’ ‘ownership’ of learning. Students should be encouraged to build on their current knowledge and skills, and share the responsibility for shaping their teaching and learning.

The establishment of a supportive and inclusive learning environment should be evident from the onset of any teaching activity [20, 21]. Without it, students are less willing to participate and actively engage in the learning experiences offered. Small group teaching offers an effective mechanism to facilitate IPE in the classroom. The benefits of small group teaching include increased teaching flexibility, differentiation for student learning, increased student engagement, and active student involvement [20, 21], providing a more independent approach to learning. Close interaction of group members provides a community like environment, social interaction, and a shared sense of identity, leading to a more meaningful learning experience [20, 21].

Facilitating interprofessional groups of students can be rewarding as well as challenging, as the diverse group of students look to the facilitator for guidance. While advice and guidance may be offered it can be difficult to remove the discipline specific ‘hat’, and consider all health professional responses. Key to good facilitation is a shared depth of disciplinary knowledge around student learning outcomes, and a focus on the interprofessional collaborative outcomes. Other elements essential to facilitation of IPE activities include demonstration of appreciation and respect for the roles of other health professionals, promotion of team formation and conflict resolution, and insight into one’s own professional practice [17]. Additionally, the use of online media to deliver IPE is becoming increasingly prevalent [22, 23]. This reflects the adjustment needed to overcome a range of timetabling and geographical difficulties associated with the face-to-face delivery of IPE [22, 23]. Although there is much research needed in this area, a recent review highlighted the need for facilitators to be proactive in guiding learners to share their professional perspectives on the online IPE discussion boards [22].

#### Facilitating IPE in the clinical setting

Facilitators often feel they are capable and well prepared to teach students of their own profession, but not those of other health professions. Egan-Lee et al. (2011) state that facilitating interprofessional groups of students in the clinical setting requires a specific skill set, and incorporates a range of attributes, including: confidence, flexibility in managing professional conflict, and a commitment to IPE [24]. Tips to encourage trust between health professions [25–27]:

- Trust develops over time, be patient and work on the development of interpersonal relationships
- Use students’ names, not role or location
- A disagreement should not be interpreted as disrespect, just differing opinion
- Offer your skills and knowledge, with trust developing through your successes.

Table 3 provides ‘ten tips’ to assist facilitation of interprofessional learning and the building of positive team function’ [7, 13, 20, 21, 26–33].

**Table 3:** Ten tips to assist facilitation of interprofessional learning and the building of positive team function

**Structured, early orientation to IPL**	Early participation in IPE activities promotes recognition of the need for effective communication within healthcare teams, and better prepares students for interprofessional practice.
**Role of other professions**	Ensure you have a good understanding of the role of each profession.
**Questioning**	Allow discussion time, and elicit answers from the students, rather than giving answers yourself. For example, *“What evidence supports your claim?”* Use a reflective approach, with probing questions that enable the development of students’ problem solving and clinical reasoning skills.
**Focus on the needs of patients**	Assist in the breakdown of hierarchical barriers by focussing on patient needs and patient safety.
**Trust**	Encourage the building of relationships and trust, between both the facilitator and students, and also within student teams. Trust is established through ongoing professional and personal interactions [25].
**Flipped classroom approach**	The flipped classroom approach to interprofessional education has many benefits. It encourages a ‘level playing field’, where all students are provided with the same pre-class information, and attend class with this assumed knowledge. This frees up in-class time for student-centred learning, where the facilitator is free to support the knowledge and skills development of students.
**Be a facilitator, not a lecturer**	You are not a content expert in every discipline. Although you may be more accustomed to delivering content, rather than facilitating discussion, it is important to facilitate, and not lecture. Try to follow the 90:10 rule: listen for 90% of the time, and talk for 10% [26].
**Peer learning**	Encourage peer teaching and learning. Students are closer to each other in terms of knowledge and skills, and are likely to have a greater understanding than tutors regarding concepts their peers are struggling with. They are sometimes better than faculty at teaching concepts to one another [27].
**Review and reflect**	Since interprofessional activities are normally focussed on a health topic, or patient case, students may not realise how much they have learnt about each other’s professions. For this reason, it is recommended that you review and make the interprofessional concepts explicit at the end of class, to help students recognise the outcomes, and their achievements.
**Assessment and Feedback**	There are many available interprofessional competency frameworks to draw from in designing assessment activities [5, 8, 28]. The use of peer assessment and peer feedback is well suited to interprofessional activities, promoting self-assessment and reflection on one’s own work. However, peer assessment may be viewed negatively if the process is not transparent, with clear assessment criteria [29].

#### Key challenges of IPE facilitation

The role of the facilitator is central in mediating group dynamics, although team members also have opportunities to influence and diffuse potential issues that may arise in small teams. Facilitators often find it a challenge to support ‘teamwork’, as with student centred learning, facilitators are required to support the team, but also need to allow them to work independently [26]. Common barriers to effective teamwork include [33]:

- Lack of communication skills
- Differing professional cultures
- Traditional hierarchies and assumed leadership
- ‘Role blurring’, confusion over boundaries and responsibilities.

### Planned IPE activities

Some activity designs are more effective and better suited to the delivery of IPE. Small group teaching is an effective method for facilitating interprofessional student groups and preparation is essential for effective student learning [34]. Examples of methods of teaching used in design of interprofessional activities include Team-based learning (TBL), simulation and student led interprofessional clinics [35, 36]. In particular, in the junior years, TBL has the capacity to draw participants’ attention to the process of learning, and has been correlated with encouraging better teamwork skills and improved communication [35].

### Peer teaching in IPE

Within IPE activities, peer teaching provides a form of student interaction facilitated within formal professional contexts [20, 21]. Learning in this context provides a process of socialisation, where students have the opportunity to share their experiences within their own discipline, with students from other disciplines. The social and cognitive congruence of students provides a quality that is difficult to emulate, as they learn from each other. IPE activities provide students with opportunities to familiarise themselves with the different language and tasks of each others’ professions. Concurrently, this process contributes to the development of students’ own professional identity, and their understanding of different professional responsibilities [29].

### Assessment and feedback during IPE activities

The provision of accurate, timely feedback to learners on their progress towards achievement of IPE outcomes is a critical component of health professional education programs [37]. Feedback should be seen as an active process that emphasises the agency of the learner as an active seeker of feedback on the basis of which they can improve their performance. Giving and receiving peer feedback within the interprofessional context can be powerful. The views of health professionals outside of one’s own discipline is often meaningful, increasing self-reflection. Multidisciplinary feedback has the ability to promote reflection on communication and the use of terminology [24].

### Evaluating the implementation of IPE activities

A Cochrane systematic review provides evidence that IPE evaluation and research lacks rigorous design, and to date has not effectively provided insight into how IPE affects change in healthcare processes and patient outcomes [38]. The authors suggest research be explicitly focused on IPE, include comparative studies, and large sample sizes. Reeves et al. (2015) suggest that high quality evaluations of interprofessional education include the following steps [38]:

1. Plan and consider the evaluation early on during curriculum development
2. Have a clear purpose for the evaluation and form concise evaluation questions
3. Have an understanding of the intended stakeholders and the learning outcomes
4. Consider the use of a theoretical perspective to strengthen the evaluation
5. Use an evaluation model that adopts a comprehensive approach, and explores the processes related to the learning activity
6. Select an evaluation design that reflects the research question, considering whether quantitative or qualitative evaluation design, or a mixed methods evaluation is required.

## Conclusion

Although IPE is an integral feature of forward thinking university health education programs, it is often raised as deficit, with many existing challenges, including adequate curriculum space and funding [39]. Planning, design and facilitation of interprofessional learning is challenging, but achievable through the creation of authentic IPE activities for health professional students. Early training and experiences of IPE have the potential to lead to improved leadership, collaboration and communication between healthcare teams, ultimately improving patient safety [39–41].

### Take-home message

**Table:** 

• Early participation in IPE activities promotes recognition of the need for effective communication between different health professionals, and helps prepare students for professional practice • Facilitation of interprofessional student groups often requires more rigorous preparation and guidance • The ‘flipped classroom’ approach to interprofessional education helps to ensure a ‘level playing field’ for students from different disciplines, and helps free up in-class time	

## Data Availability

Not applicable.

## Abbreviations

IPE: Interprofessional education
WHO: World Health Organisation
TBL: Team-based learning
OSCE: Objective Structured Clinical Examinations
